# Supplementary material for: COVID-19 misinformation: Mere harmless delusions or much more? A knowledge and attitude cross-sectional study among the general public residing in Jordan
Source: PLoS One. 2020 Dec 3;15(12):e0243264. doi: 10.1371/journal.pone.0243264 (PMC7714217; doi:10.1371/journal.pone.0243264)
Supplement: S1 Appendix — (PDF) [file pone.0243264.s001.pdf]

S1 Appendix

Consent Form and Questionnaire Translated to English (the original form in Arabic is provided below).

Coronavirus Disease 2019 (COVID-19): Level of knowledge in Jordan

This questionnaire was prepared to measure the extent of knowledge and awareness of the population of Jordan about the threats posed by the novel coronavirus.

The information provided to you through this questionnaire will be used for research purposes only.

Participation in this survey is completely voluntary.

Please answer the questionnaire through your current knowledge of the disease, without having to refer to any source or site related to the topic.

Thank you very much for agreeing to participate in this survey

---

Age:

……………………

Nationality:

- ☐ Jordanian
- ☐ Non-Jordanian

Gender

- ☐ Male
- ☐ Female

In which governorate do you live in?

- ☐ Amman
- ☐ Zarqa
- ☐ Irbid
- ☐ Ajlun
- ☐ Aqaba
- ☐ Madaba
- ☐ Mafraq
- ☐ Ma`an
- ☐ Jarash
- ☐ Tafilah
- ☐ Karak
- ☐ Balqa

Marital Status

- ☐ Single
- ☐ Married
- ☐ Divorced
- ☐ Widowed

Family monthly income:

- ☐ Less than 500 JDs
- ☐ Between 500 and 1000 JDs
- ☐ More than 1000 JDs

Educational level:

- ☐ Highschool or less
- ☐ Diploma
- ☐ Bachelors
- ☐ Masters
- ☐ PhD

Are you a smoker?

- ☐ Yes
- ☐ No

Do you suffer from any chronic diseases (like Diabetes, Hypertension, or heart conditions)?

- ☐ Yes
- ☐ No

Is COVID-19 a dangerous disease?

- ☐ Not dangerous
- ☐ Moderately dangerous
- ☐ Very dangerous

Are you adhering to government quarantine rules and staying home?

- ☐ Yes
- ☐ No

Do you think that the quarantine gave you the opportunity to spend a quality time with your family?

- ☐ Yes
- ☐ No

Do you feel annoyed by your inability to attend places of worship (mosques and churches)?

- ☐ Yes
- ☐ No

Knowledge about COVID-19

	True	False
Fever is one of the clinical signs of the disease	☐	☐
Coughing is one of the clinical signs of the disease	☐	☐
COVID-19 can cause diarrhea and vomiting.	☐	☐
Shortness of breath is a symptom of the disease.	☐	☐
The virus can be transmitted through touching infected surfaces.	☐	☐
The virus can remain viable on surfaces for few hours.	☐	☐
The virus can be transmitted through close contact with an infected person.	☐	☐
The virus can be transmitted through blood.	☐	☐
The coronavirus infection can be treated using an antibiotic.	☐	☐
There is an available vaccine for COVID-19.	☐	☐
The virus can be killed by home remedies like garlic, onions, and ginger.	☐	☐
The summer heat can kill the COVID-19 virus.	☐	☐
Re-infection by COVID-19 is possible.	☐	☐

What is the main source of your knowledge about COVID-19?

☐ Ministry of Health official website

☐ Scientific journals

☐ Medical Doctors

☐ Television programs and news releases

☐ Social media (Facebook, Instagram, Twitter, WhatsApp)

If you answered yes on social media, which platform do you mainly receive your information from?

- Facebook
- Instagram
- Twitter
- WhatsApp
- YouTube

Do you think the COVID-19 outbreak is part of a global conspiracy theory?

- Yes
- No

Do you think that 5G (5th Generation) networks are spreading COVID-19?

- Yes
- No

Do you think COVID-19 is part of a biological warfare?

- Yes
- No

Do you think that COVID-19 is a spiritual test from God?

- Yes
- No

Answer the questions regarding how you have been feeling the past 2 weeks of quarantine.

	Not at all (0)	Several days (1)	More than half the days (2)	Nearly every day (3)
1. Are you feeling nervous or anxious?	☐	☐	☐	☐
2. You have trouble relaxing and feeling comfortable	☐	☐	☐	☐
3. You worry a lot about the numbers of people infected with the virus.	☐	☐	☐	☐
4. Inability to carry on with daily work.	☐	☐	☐	☐
5. Being so restless and unable to stop thinking	☐	☐	☐	☐
6. Becoming easily annoyed or irritable during the quarantine period.	☐	☐	☐	☐
7. Feeling scared that this pandemic might turn into a global catastrophe.	☐	☐	☐	☐

Thank you

مرض فيروس كورونا ٢٠١٩: مستوى المعرفة في الأردن

تم اعداد هذا الاستبيان لقياس مدى المعرفة في الاردن حول مخاطر فيروس الكورونا المستجد

سيتم استخدام المعلومات المقدمة لك من خلال هذا الاستبيان لأغراض بحثية فقط

المشاركة في هذه الإستبانة طوعية تمامًا

شكرا جزيلا للموافقة على المشاركة في هذا الاستطلاع.

العمر: ....

الجنسية:

- ☐ أردني
- ☐ غير أردني

الجنس:

- ☐ ذكر
- ☐ انثى

في أي محافظة تعيش:

- ☐ محافظة العاصمة عمّان
- ☐ محافظة الزرقاء
- ☐ محافظة إربد
- ☐ محافظة عجلون
- ☐ محافظة العقبة
- ☐ محافظة مادبا
- ☐ محافظة المفرق
- ☐ محافظة معان
- ☐ محافظة جرش
- ☐ محافظة الطفيلة
- ☐ محافظة الكرك
- ☐ محافظة البلقاء

الحالة الاجتماعية:

- أعزب
- متزوج
- مطلق
- أرمل

الدخل الشهري للأسرة:

- أقل من 500 دينار
- بين 500 و 1000 دينار
- أكثر من 1000 دينار

المستوى التعليمي:

- توجيهي أو أقل
- دبلوم
- بكالوريوس
- ماجستير
- دكتوراه

هل أنت مدخن؟

- نعم
- لا

هل تعاني من أي أمراض مزمنة (مثل السكري، و الضغط, و امراض في القلب)

- نعم
- لا

هل فيروس كورونا المستجد فيروس خطير؟

- ليس خطيراً
- خطير إلى حد ما
- خطير جدا

هل تلتزم بقواعد الحجر الصحي الحكومي وتبقى في المنزل؟

☐ نعم

☐ لا

هل تعتقد أن الحجر المنزلي ساعدك على قضاء وقت إيجابي مع عائلتك؟

☐ نعم

☐ لا

هل أنت منزعج من عدم قدرتك على ممارسة الشعائر الدينية في أماكن العبادة (المسجد او الكنيسة)

☐ نعم

☐ لا

المعرفة حول فيروس كورونا المستجد	صح	خطأ
الحرارة من أعراض الإصابة بالفيروس	☐	☐
السعال من أعراض الإصابة بالفيروس	☐	☐
الإسهال والقيء من أعراض الإصابة بالفيروس	☐	☐
ضيق في التنفس من أعراض الإصابة بالفيروس	☐	☐
ينتقل الفيروس من خلال ملامسة الأسطح	☐	☐
يعيش الفيروس على الأسطح لساعات قليلة فقط	☐	☐
ينتقل الفيروس بالإتصال المباشر مع الشخص المصاب	☐	☐
يمكن للفيروس الانتقال عن طريق الدم	☐	☐
يمكن علاج عدوى فيروس كورونا باستخدام مضاد حيوي	☐	☐
يوجد لقاح متوفر لفيروس كورونا	☐	☐
يمكن القضاء على الفيروس باستخدام الثوم، والبصل، و الزنجبيل	☐	☐
حرارة الصيف يمكن أن تقتل الفيروس	☐	☐
يمكن للشخص أن يصاب مرة أخرى بالفيروس	☐	☐

ما هو المصدر الرئيسي لمعلوماتك حول الفيروس.

- ☐ الموقع الرسمي لوزارة الصحة
- ☐ المجلات العلمية
- ☐ أطباء
- ☐ برامج التلفاز و النشرات الاخبارية
- ☐ وسائل التواصل الاجتماعي (Facebook ،Instagram ،Twitter ،WhatsApp)

إذا أجبت نعم على وسائل التواصل الاجتماعي، من أي منصة تحصل على المعلومات؟

- ☐ Facebook
- ☐ Instagram
- ☐ Twitter
- ☐ WhatsApp
- ☐ YouTube

هل تعتقد أن الفيروس جزء من نظرية المؤامرة العالمية؟

- ☐ نعم
- ☐ لا

هل تعتقد أن شبكات G5 (الجيل الخامس) تنشر الفيروس؟

- ☐ نعم
- ☐ لا

هل تعتقد أن فيروس كورونا جزء من حرب بيولوجية؟

- ☐ نعم
- ☐ لا

هل تعتقد أن مرض فيروس كورونا (كوفيد-19) هو ابتلاء أو امتحان من الله للبشر؟

- ☐ نعم
- ☐ لا

أجب عن الأسئلة بما كنت قد شعرت به خلال الأسبوعين الماضيين من الحجر الصحي

	لا على الاطلاق	عدة أيام	أكثر من نصف الأيام	كل يوم تقريبا
هل تشعر بالتوتر أو القلق ؟	☐	☐	☐	☐
لديك مشكلة في الاسترخاء و الشعور بالراحة.	☐	☐	☐	☐
تقلق كثيرا بشأن أعداد المصابين بالفيروس.	☐	☐	☐	☐
عدم القدرة على الاستمرار بالأعمال اليومية.	☐	☐	☐	☐
عدم التوقف عن التفكير	☐	☐	☐	☐
تشعر بالانزعاج بسرعة في فترة الحجر	☐	☐	☐	☐
الشعور بالخوف من تحول الوباء الى كارثة عالمية.	☐	☐	☐	☐

شكرا
