# Supplementary material for: COVID-19 misinformation: Mere harmless delusions or much more? A knowledge and attitude cross-sectional study among the general public residing in Jordan
Source: PLoS One. 2020 Dec 3;15(12):e0243264. doi: 10.1371/journal.pone.0243264 (PMC7714217; doi:10.1371/journal.pone.0243264)
Supplement: S2 Appendix — (PDF) [file pone.0243264.s002.pdf]

S2 Appendix

Supplementary Tables (Additional Results)

Table S1. Attitude of the study participants to quarantine measures stratified by socio-demographic variables.

	Survey question	Are you adhering to government quarantine rules and staying home?				Do you think that the quarantine helped you spend a quality time with your family?				Do you feel annoyed regarding your inability to practice prayers in places of worship (mosque or church)?			
**Variable**		**YES**		**NO**		**YES**		**NO**		**YES**		**NO**	
		**N**	**%**	**N**	**%**	**N**	**%**	**N**	**%**	**N**	**%**	**N**	**%**
**Gender**	*Male*	713	95.7%	32	4.3%	614	82.9%	127	17.1%	523	70.3%	221	29.7%
	Female	2318	98.7%	31	1.3%	2031	86.4%	321	13.6%	1561	66.3%	794	33.7%
**Nationality**	*Jordanian*	2824	97.9%	61	2.1%	2482	86.1%	402	13.9%	1953	67.6%	936	32.4%
	non-Jordanian	219	98.6%	3	1.4%	175	78.8%	47	21.2%	139	62.3%	84	37.7%
**Residence place**	*Amman*	1806	98.3%	32	1.7%	1575	85.6%	266	14.4%	1176	63.8%	667	36.2%
	Outside Amman	1193	97.5%	30	2.5%	1039	85.2%	181	14.8%	882	72.0%	343	28.0%
**Monthly income**	*Less than 500 JOD*	1200	97.6%	29	2.4%	1049	85.5%	178	14.5%	888	72.1%	343	27.9%
	500-1000 JOD	1161	98.6%	16	1.4%	1022	86.7%	157	13.3%	810	68.7%	369	31.3%
	More than 1000 JOD	647	97.1%	19	2.9%	556	83.5%	110	16.5%	370	55.4%	298	44.6%
**Educational level**	*High school or less*	482	97.6%	12	2.4%	421	85.7%	70	14.3%	384	78.0%	108	22.0%
	Undergraduate degree	2262	98.1%	43	1.9%	1954	84.7%	353	15.3%	1536	66.5%	774	33.5%
	Postgraduate degree	322	97.3%	9	2.7%	301	90.7%	31	9.3%	192	57.7%	141	42.3%

Table S2. Belief in conspiracy in relation to socio-demographic variables among the study participants

	Survey question	Do you think the COVID-19 pandemic is part of a global conspiracy theory?				Do you believe that 5G networks are helping in spread of COVID-19?				Do you think that COVID-19 is related to biological warfare?				Do you think that COVID-19 is a trial or test from God to humankind?			
**Variable**		**YES**		**NO**		**YES**		**NO**		**YES**		**NO**		**YES**		**NO**	
		**N**	**%**	**N**	**%**	**N**	**%**	**N**	**%**	**N**	**%**	**N**	**%**	**N**	**%**	**N**	**%**
**Gender**	*Male*	305	41.2%	435	58.8%	93	12.8%	631	87.2%	357	48.6%	377	51.4%	562	75.8%	179	24.2%
	Female	1175	50.1%	1171	49.9%	539	23.6%	1743	76.4%	1398	59.7%	943	40.3%	1998	85.0%	353	15.0%
**Nationality**	*Jordanian*	1397	48.5%	1484	51.5%	596	21.2%	2209	78.8%	1649	57.5%	1220	42.5%	2401	83.3%	482	16.7%
	non-Jordanian	96	43.8%	123	56.2%	43	20.0%	172	80.0%	119	54.1%	101	45.9%	172	77.1%	51	22.9%
**Capital vs others**	*Amman*	890	48.4%	947	51.6%	364	20.4%	1424	79.6%	1052	57.4%	781	42.6%	1476	80.1%	366	19.9%
	Outside Amman	581	47.7%	637	52.3%	259	21.8%	928	78.2%	691	57.1%	520	42.9%	1054	86.5%	165	13.5%
**Monthly income**	*Less than 500 JOD*	618	50.5%	605	49.5%	287	24.2%	901	75.8%	713	58.6%	503	41.4%	1086	88.4%	142	11.6%
	500-1000 JOD	566	48.2%	608	51.8%	240	20.9%	907	79.1%	695	59.3%	477	40.7%	976	82.9%	201	17.1%
	More than 1000 JOD	292	43.8%	375	56.2%	104	15.9%	550	84.1%	337	50.6%	329	49.4%	477	71.6%	189	28.4%
**Educational level**	*High school or less*	247	50.4%	243	49.6%	131	28.0%	337	72.0%	276	56.7%	211	43.3%	435	88.2%	58	11.8%
	Undergraduate degree	1116	48.5%	1183	51.5%	460	20.4%	1791	79.6%	1338	58.4%	953	41.6%	1911	82.9%	393	17.1%
	Postgraduate degree	136	40.8%	197	59.2%	50	15.5%	273	84.5%	161	48.3%	172	51.7%	244	73.5%	88	26.5%
