# Supplementary material for: COVID-19 misinformation: Mere harmless delusions or much more? A knowledge and attitude cross-sectional study among the general public residing in Jordan
Source: PLoS One. 2020 Dec 3;15(12):e0243264. doi: 10.1371/journal.pone.0243264 (PMC7714217; doi:10.1371/journal.pone.0243264)
Supplement: S3 Appendix — (PDF) [file pone.0243264.s003.pdf]

S3 Appendix

The number and percentage of item non-response in the survey.

Survey Item	Valid responses	Missing responses	Item non-response Percentage (%)
Age	**2947**	**203**	**6.4**
K-Score	**2988**	**162**	**5.1**
Anxiety score	**3035**	**115**	**3.7**
Do you believe that 5G networks are helping in spread of COVID-19?	**3053**	**97**	**3.1**
Governorates of Jordan	**3072**	**78**	**2.5**
Monthly income	**3081**	**69**	**2.2**
Gender	**3103**	**47**	**1.5**
Nationality	**3117**	**33**	**1.0**
Marital status	**3119**	**31**	**1.0**
Do you think that COVID-19 is related to biological warfare?	**3122**	**28**	**0.9**
Main Source of Knowledge about COVID-19	**3128**	**22**	**0.7**
Smoking	**3131**	**19**	**0.6**
Do you think the COVID-19 pandemic is part of a global conspiracy theory?	**3133**	**17**	**0.5**
History of chronic disease	**3136**	**14**	**0.4**
Do you think that the quarantine helped you spend a quality time with your family?	**3136**	**14**	**0.4**
Are you adhering to government quarantine rules and staying home?	**3137**	**13**	**0.4**
Educational degree	**3139**	**11**	**0.3**
Do you think that COVID-19 is a trial or test from God to humankind?	**3139**	**11**	**0.3**
Do you feel annoyed regarding your inability to practice prayers in places of worship (mosque or church)?	**3144**	**6**	**0.2**
Is COVID-19 a dangerous disease?	**3145**	**5**	**0.2**
