# Supplementary material for: COVID-19 misinformation: Mere harmless delusions or much more? A knowledge and attitude cross-sectional study among the general public residing in Jordan
Source: PLoS One. 2020 Dec 3;15(12):e0243264. doi: 10.1371/journal.pone.0243264 (PMC7714217; doi:10.1371/journal.pone.0243264)
Supplement: Attachment — Submitted filename: Rebuttal_Letter_PONE-D-20-21682R1.docx [file pone.0243264.s004.docx]

Date: 05-11-2020

Malik Sallam

School of Medicine

The University of Jordan

Amman, Jordan

Dear Dr. Flávia L. Osório

Regarding our manuscript with ID: **PONE-D-20-21682R1**, entitled “**COVID-19 misinformation: mere harmless delusions or much more? A knowledge and attitude cross-sectional study among the general public residing in Jordan**”,

We would like to thank the third reviewer for the interesting, insightful and helpful comments that helped us to improve the quality and clarity of the manuscript. In the following pages are our point-by-point responses to each of the comments raised by the third reviewer.

Please find attached a revised version of our manuscript “COVID-19 misinformation: mere harmless delusions or much more? A knowledge and attitude cross-sectional study among the general public residing in Jordan”. The revisions were highlighted using the "Track Changes" function in the manuscript file. A clean version of the revised manuscript is submitted as well.

We hope that the revisions in the manuscript and our accompanying responses will be sufficient to make our manuscript suitable for publication in *PLoS One*.

Sincerely, and on behalf of the co-authors

Malik Sallam. MD, PhD

**Responses to the comments of Reviewer #3**

**Reviewer #3: This is an interesting paper that explores knowledge, attitudes, and misinformation about COVID-19 and its effects on anxiety levels among adults in Jordan. Sources of information about the virus are also explored. The results of this paper are very important to highlight the consequences of misinformation so that the delivery of timely and accurate information is prioritized as pandemic management strategies are updated. However, the manuscript will need restructuring to better guide the reader through the paper. In particular, the authors should ensure that each analysis and writing decision always links back to the original research aims.**

**Below are more detailed comments and recommendations:**

**Major issues**

**Introduction**

**1. The introduction would benefit from supplementation and restructuring in order to provide the necessary background to frame the importance of this research, as well as to contextualize the setting in which this research was undertaken. I would suggest the following structure: (1) Global impact of COVID-19 (which the authors have already included); (2) Proper conceptualization of misinformation (e.g. definition, how it spreads) and its consequences (e.g. deaths in Iran due to false belief that ingesting methanol kills the virus); (3) Context of COVID-19 in Jordan (some details have been given in the response to reviewer 1’s last comment, but would be helpful if this were expanded upon and included in the main manuscript); (4) Why examining anxiety level is warranted/important, since it is one of the key outcome variables.**

***Response:*** Based on the reviewer’s suggestion, we added the following paragraph to define and show the effects of misinformation about COVID-19 at the global level as follows (in the clean version of the manuscript):

Page 3, lines 64-69: “The World Health Organization (WHO) has declared early on during the course of COVID-19 pandemic the existence of an accompanying “infodemic” [16]. This infodemic was defined as “an over-abundance of information – some accurate and some not – that makes it hard for people to find trustworthy sources and reliable guidance when they need it” [16]. Inaccurate or false information that are communicated regardless of the deception intent is termed “misinformation” [17, 18]. This includes the circulation of conspiracy theories that prevail at times of fear and uncertainty [19].”

In addition, we supplemented the paragraph on COVID-19 in Jordan to elaborate on the rapid escalation of cases since late August as follows (in the clean version of the manuscript):

Page 4, lines 78-83: “In Jordan, strict governmental-issued infection control measures that included wide lockdowns, curfew, mask and social distancing enforcement, and prohibition of large gatherings were helpful in delaying the first wave of COVID-19 epidemic in the country. However, these measures can be viewed currently as “delaying the inevitable”, since the number of daily diagnosed cases of COVID-19 escalated rapidly from late August 2020, to reach more than 60,000 active cases by the end of October 2020 [28].”

Finally, we included a paragraph to show the importance of studying the anxiety levels in time of crisis/emergency as follows (in the clean version of the manuscript):

Page 4, lines 87-88: “Studying anxiety is of prime importance as well, since it may drive the public behavior and attitude towards the infection control and mitigation measures [29, 30].”

**2. The objectives of the study are very clearly stated in lines 76-79 (clean version), which is great to see. These should guide the way that the subsequent sections of the paper are structured, as I will mention in a few comments below.**

***Response:*** We would like to thank the reviewer for the comment. We will address the re-structure of subsequent sections according to the reviewer’s suggestions in the comments below (comment #12).

**Methods**

**3. (Statistical analysis) While I see the author’s use of regression as a response to a comment from reviewer 1, it is not well justified why the most “complex” statistical approach was employed on belief in conspiracy as an outcome, since this is not one of the main research objectives. As the aim of the study is to evaluate knowledge, attitude and misinformation *on anxiety level*, if regression is used, it should be on this outcome. If the authors choose to retain their regression analyses on belief in conspiracy, why this approach was taken for this outcome in particular needs to be justified.**

***Response:*** We would like to thank the reviewer for raising this important issue that helped us to clarify our aim from conducting such analysis in response to reviewer 1. We agree that the use of belief in conspiracy as the outcome would not reflect our major aims (effect of misinformation on anxiety). In addition, we agree that the use of such a complex statistical model might not be appropriate for such an aim, in view of our previous analysis that was shown in the results section (Page 15, lines 319-323 of the clean version: “Higher anxiety scores were found among those who believed that COVID-19 is related to conspiracy (9.7 vs. 8.7; p<0.001; M-W), biological warfare believers (9.6 vs. 8.6, p<0.001; M-W), those who believed in the role of 5G networks in facilitating COVID-19 spread (10.3 vs. 8.9, p<0.001; M-W), and those who believed that the pandemic is a spiritual divine test (9.4 vs. 8.0, p<0.001; M-W, Fig 6)”. Thus, we omitted this part from the revised manuscript. However, to address the issue raised by reviewer 1, we re-phrased some strong statements in the abstract and discussion sections as follows:

In the abstract (Page 2, lines 37-40): “Misinformation about the origin of the pandemic (being part of a conspiracy, biologic warfare and the 5G networks role) was also associated with higher anxiety levels. Social media platforms, TV and news releases were the most common sources of information about the pandemic” Instead of “Misinformation about the origin of the pandemic (being part of a conspiracy, biologic warfare and the 5G networks role) was also associated with higher anxiety and lower knowledge about the disease”.

In the discussion section (Page 18, lines 387-388): “The results of the current study showed some harmful effects of belief in conspiracy in relation to higher anxiety levels” instead of “The results of the current study clearly delineate the existence of an association between misinformation about COVID-19 and the combination of higher anxiety and lower knowledge about the disease among the public in Jordan”.

In addition, we deleted the following paragraphs (in the revised highlighted manuscript): Page 8, lines 165-168: “To analyze the variables associated with higher likelihood of believing in the role of conspiracy in the origin of the pandemic, we conducted multinomial logistic regression analysis using the following variables (age, gender, nationality, monthly income, educational level, K-score and anxiety score).” Page 16, lines 320-323: “Using multinomial logistic regression analysis, we found an association of belief in conspiracy regarding the origin of the pandemic with a lower K-score (p<0.001), a higher anxiety score (p=0.006), female gender (p<0.001), and lower monthly income (p=0.029).”

**Results
4. While the level of detail the authors have included in their results is commendable, the sheer volume of results may be overwhelming for the reader. I suggest picking out results that will be expanded on in the discussion instead of reporting on each finding individually, and in particular, being more selective about which demographic associations to report.**

***Response:*** Although we agree with the reviewer regarding the length of the results section, but we still believe that we included the most significant results only. The results section might appear more succinct once the figures, figure legends and tables are formatted according the final editing by the journal. We hope that the reviewer would agree with us regarding our motivation to keep the results section in the current length after re-organization as suggested.

**5. The sections of the results should be restructured so that the research questions, as stated in the introduction, are answered in sequence: (1) Knowledge, attitudes, and misinformation; (2) The effects of (1) on anxiety; (3) COVID-19 information sources.**

***Response:*** Based on the reviewer’s suggestion and to keep the flow of text in the same order based on the objectives of the study, we re-organized the results section as follows:

- Characteristics of the study population
- COVID-19 knowledge
- Perception of COVID-19 danger and attitude towards quarantine
- Beliefs and misinformation about COVID-19 origin

1. Is COVID-19 part of a global conspiracy?
2. Is COVID-19 part of a biological warfare?
3. Do 5G networks have a role in COVID-19 spread?
4. Is COVID-19 a spiritual divine test or trial?

- Anxiety regarding COVID-19
- The main media and other sources of information about the pandemic

**6. If regression analysis is used, it is important that its results are displayed in a table, and for coefficient estimates to be reported in the main text, in addition to the significance levels that are currently present in the manuscript.**

***Response:*** Please refer our previous reply to comment #3.

**Discussion
7. Discussions about the belief in conspiracy theories and anxiety levels were addressed separately, and as I understood, mainly highlighted prevalence and demographic associations, respectively. However, this discussion piece should be reworked to address effects of misinformation on anxiety in order to answer the second research objective.**

***Response:*** Based on the reviewer’s suggestion, we added to our previous discussion the following statements (clean version):

Pages 18-19, lines 393-399: “The current climate of fear and uncertainty seems as a fertile soil from which conspiracy beliefs are born and thrive [50, 51]. Thus, the high prevalence of inaccurate beliefs about COVID-19 origins seems a plausible result. In addition, the link that was demonstrated between higher anxiety levels and conspiracy belief in this study, is not unique and can augment the previous evidence that such belief is harmful [27, 52, 53].”

“In particular, a recent study showed that belief in conspiracy is a predictor of distress and anxiety among health-care workers [54]. Another study found that such belief was associated with the presence of depression or distress [55].”

**Conclusion**

**8. The key takeaway message needs to be more clearly highlighted. Based on the title of the paper, it would seem that the main message is: not only has this paper shown that misinformation is prevalent, but that it contributes to higher anxiety levels (i.e. more than “harmless delusions).**

***Response:*** We would like the reviewer for helping us to strengthen and clarify the conclusions of this research. Thus, we added the following statement to the conclusions section (clean version):

Page 21, lines 468-460: “Belief in the role of conspiracies, biological warfare, and 5G networks in the origin and spread of the disease was prevalent in this study and was also associated with a higher level of anxiety, which indicates that COVID-19 misinformation may have harmful effects among the general public.”

**9. Having framed the issue that this study has revealed and to round off the conclusion, the authors could consider highlighting another important finding as a solution – that knowledge from reliable information sources seems to reduce this anxiety.**

***Response:*** We also would like to thank the reviewer for this insightful comment and based on the reviewer’s suggestion, we added the following statement to the conclusions section (clean version):

Pages 21-22, lines 472-476: “Our results indicated that the reliance on reliable sources to get information on the current pandemic (e.g. scientific journals and medical doctors) was associated with lower levels of anxiety. This demonstrates the significant role that should be played by the scientific community and clinicians to address the gaps in knowledge and to correct misinformation among the general public.”

**Minor issues**

**Introduction**

**10. The use of the correct tense needs to be checked for consistency and/or appropriateness throughout the paper (e.g. “The entire world is facing an unprecedented challenge…” (line 46, clean version) vs. “Coronavirus disease (COVID-19) resulted in…” (line 47-48, clean version))**

***Response:*** Based on the reviewer’s comments, we made the following changes (in the clean version of the manuscript):

-Page 3, line 46: “The entire world faced an unprecedented challenge” instead of “The entire world is facing an unprecedented challenge”.

-Page 3, line 49: “The public was left in a state of disarray” instead of “The public is left in a state of disarray”

**Methods
11. The authors may consider removing the following sentences as this information is already present elsewhere in the methods: “Participation in the study was voluntary and an informed consent was included. The questionnaire was sent through Facebook, WhatsApp, Twitter and other social media platforms.” (lines 87-89, clean version).**

***Response:*** Based on the reviewer’s comment, we omitted the following sentence from the study design and description of the questionnaire part of the methods section: “The questionnaire was sent through Facebook, WhatsApp, Twitter and other social media platforms” and from the ethical permission paragraph of the methods section: “Participation in the study was voluntary and anonymous.”

**12. The methods section would also benefit from restructuring so that it is more intuitive for the reader to follow. The first paragraph is great, and I would recommend describing the questionnaire sections in the same sequence that they are mentioned in 93-94 (clean version): knowledge, attitude, misinformation, information sources, anxiety, and then sociodemographic variables. The descriptions of ethical permission and statistical approach can then follow.**

***Response:*** Based on the reviewer’s suggestion, we re-organized the methods section as follows (in the clean version):

- Study design and description of the questionnaire
- COVID-19 knowledge score (K-score) calculation
- Assessment of the anxiety score
- Source of information about COVID-19
- Ethical permission
- Statistical analysis.

**13. In order to avoid confusion about what a “valid” K-/anxiety score may mean (e.g. that the measures were constructed from a validated scale), lines 121/122 and 129/130 (clean version) could be reworded to something like "****Individual K-scores were considered valid and included in the analyses if the participant provided responses to all 13 items”.**

***Response:*** We appreciate the kind help of the reviewer that enabled us to clarify this statement. And based on that, we changed the two sentences as follows (clean version):

Page 6, lines 126-127: “Individual K-scores were considered valid and included in the analyses if the participant provided responses to all 13 items.”

Page 6, lines 135-136: “Individual anxiety-scores were considered valid and included in the analyses if the participant provided responses to all seven items.”

**14. It is important to describe what “as appropriate” (line 135, clean version) means – which tests were used, for what, and why?**

***Response:*** Based on the reviewer’s suggestion, we added the following sentences to clarify the statistical tests used in the study (in the clean version):

Page 7, lines 153-157: “We used the chi-squared (χ^2^) test to evaluate the significance of relationships between categorical variables. For continuous variables (e.g. age), we used the Mann-Whitney *U* (M-W) to compare the mean among two independent groups, and the Kruskal Wallis (K-W) test to compare the mean among more than two independent groups.”

**Results**

**15. Similar to reviewer 1, I am also confused about the 1.8% margin of error. The response the authors provided to the reviewer would be helpful to include in the manuscript, and how this margin of error was calculated should also be described.**

***Response:*** Based on the reviewers’ comment, we added the following statement to the results section (clean version):

Page 8, lines 163-165: “This resulted in a minimum of 1.8% margin of error (an expression of how much the response of the study sample is representative of the general population)”.

The calculation was based on the online website (CheckMarket: https://www.checkmarket.com/sample-size-calculator/?fbclid=IwAR0DZS_iZnJpXTv76LXR4T5BFqE6qVGjViAtP2zy-uQAtiJ1gRBWeaEk-XQ), which has already been cited in our original submission: Reference No. 32 in the clean version: CheckMarket (2020) Calculate sample size margin of error.

**16. The following sentence is incomplete: “While those who thought that the quarantine gave them an opportunity to spend a quality time with their families had a lower mean anxiety score (8.9 vs. 10.8, p<0.001; M-W).” (line 221-222, clean version)**

***Response:*** We would like to thank the reviewer for correcting this error. The sentence was corrected into (in the clean version):

Page 15, lines 316-317: “The participants who thought that the quarantine gave them an opportunity to spend a quality time with their families had a lower mean anxiety score (8.9 vs. 10.8, p<0.001; M-W).”

**17. The following sentence needs to be revised for clarity: “Older participants perceived the disease…” (lines 243-246, clean version)**

***Response:*** Based on the reviewer’s suggestion, we revised the statement as follows (clean version):

Page 11, lines 236-238: “Older participants felt the disease as more dangerous compared to younger participants (40.6% vs. 31.7%), p<0.001; χ^2^, Table 2).”

**18. The authors may consider whether gender (men/women) or sex (female/male) is being examined in the study and use the appropriate language consistently throughout the paper.**

***Response:*** Based on the reviewer’s suggestion, we used sex (female/male) instead of gender (men/women) throughout the manuscript as follows (clean version): Page 5, line 107; Page 9, Table 1; Page 13, Table 2; Page 15, line 311; Page 20, line 436.

**19. Some results that are described are not presented within the tables or figures (e.g. spending time with family, demographic associations with conspiracy beliefs or information sources). Even if these results are not the main focus of the paper, if they are mentioned in the body of the text, they should at least be included as supplementary material.**

***Response:*** Based on the reviewer’s suggestion, we added a supplementary file (S2 File: Supplementary Tables [Additional Results]). This file included two tables on the attitude of the study participants to quarantine measures stratified by socio-demographic variables and on the Belief in conspiracy in relation to socio-demographic variables among the study participants.

**20. The percentages reported for each source of information (from line 318, clean version), at first glance, appears quite low. From the questionnaire, I see that participants were asked to select their primary (vs. common) source of info and only allowed to select one option? This should be mentioned because it is more intuitive that people would consult more than one information source, and the phrasing “the most common source…” could be misleading.**

***Response:*** We would like the reviewer for this comment that allowed us to clarify this issue. The questionnaire was an electronic online survey and for the main source of information, we allowed one choice to be selected. We added this part to the methods section (clean version)

Page 6, lines 138-143:

“Source of information about COVID-19

To study the most common sources of COVID-19 information, we allowed the participants to select a single option out of the following choices: Ministry of Health official website, scientific journals, medical doctors, television programs and news releases, or social media platforms. If they selected social media, another single option was required to be answered (Facebook, Instagram, Twitter, or WhatsApp, S1 Appendix).”

**Discussion**

**21. The following sentence needs to be revised for clarity: “More than 87% correct responses were found for eight items and more than 63% correct responses for eleven items out of thirteen total items that were used to assess COVID-19 knowledge in this study” (lines 349-351, clean version)**

***Response:*** Based on the reviewer’s comment and to help increasing the clarity of the statement, we re-phrased the paragraph as follows (clean version):

Page 18, lines 367-370: “This satisfactory knowledge was shown by the percentage of correct answers in response to the total of thirteen items that were used to assess COVID-19 knowledge in this study. More than 87% correct responses were found for eight items and more than 63% correct responses for eleven items.”

**22. The authors should consider acknowledging the emergence of more recent evidence that COVID-19 reinfection may be possible (lines 356-357, clean version).**

***Response:*** Based on the reviewer’s comment, we added the following statement and a couple of references to clarify this issue (clean version):

Page 18, lines 375-377: “For the possibility of re-infection, the current evidence points to unlikely occurrence of such a phenomenon despite the need for more research tackling this aim in light of increasing reports on its genuine occurrence [35-41].”

**23. The sentence in lines 365-367 (clean version) should be revised. As it currently reads, it seems like the there is an association between misinformation and anxiety/knowledge as a combined item.**

***Response:*** Based on the reviewer’s comment and in light of reviewer 1 previous comments, we edited the sentence as follows (clean version):

Page 18, lines 385-386: “The results of the current study showed some harmful effects of belief in conspiracy in relation to lower knowledge about the disease and higher anxiety levels.”

**24. Given the length of the discussion, again, I would suggest restructuring this sentence to follow the sequence of the research objectives to make it easier to follow.**

***Response:*** We would like to thank the reviewer for the suggestion, but we prefer to keep the discussion in the current format and hopefully the reviewer would agree on that after reading the clean version of the manuscript.

**25. Could the authors expand on why they think study validity may be a limitation?**

***Response:*** We included this statement to clarify that minor modification of some COVID-19 knowledge items should be taken into consideration upon evaluating the overall knowledge about the disease. Thus, to clarify this issue, we added the following sentence in the limitations section (clean version):

Pages 20-21, lines 443-444: “The study validity can be another limitation in relation to K-score calculation, despite having a majority of items adopted from previously published work [45]”.

**26. The table of “Item non-response in the survey” provided in the reviewer response could be added as supplementary material as evidence to support the claim made in lines 425-427 (clean version).**

***Response:*** Based on the reviewer’s suggestion, we added the following supplementary file (S3 Appendix) entitled “Table on the number and percentage of item non-response in the survey.”

Clean version, Page 21, line 448: “However, we believe that the later analysis did not result in severe bias considering that item non-response did not exceed 1.0% for the majority of survey items (S3 Appendix).”

**Conclusion**

**27. The phase “public embrace of conspiracy” (line 434, clean version) is a bit strong. It may be more accurate to say that the** **prevalence of beliefs in different conspiracy theories was investigated.**

***Response:*** We would like to thank the reviewer for the comment and based on that, we changed the sentence as follows (clean version):

Page 21, lines 453-455: “In the current research, we inspected knowledge of COVID-19 at a country level, with a special focus on the prevalence of belief in different conspiracy theories regarding the origin of the pandemic.”

**28. It seems like there may be a missing word/description after “Focused awareness” (line 441, clean version).**

***Response:*** Based on the reviewer’s comment, we added the word “campaigns” to the sentence as follows (clean version):

Page 21, line 462: “Focused awareness campaigns and proper delivery of correct information is mandatory, particularly for this group to reduce the negative impact of the pandemic on their lives.”

Finally, we are deeply grateful for the thorough, insightful and comprehensive review made by the reviewers that helped us greatly in refining our research.
